# Enhancing Electron Transfer Efficiency in Microbial Fuel Cells Through Gold Nanoparticle Modification of *Saccharomyces cerevisiae*

**DOI:** 10.3390/microorganisms13081938

**Published:** 2025-08-20

**Authors:** Teresė Kondrotaitė-Intė, Antanas Zinovičius, Domas Pirštelis, Inga Morkvėnaitė

**Affiliations:** 1 Department of Mechanical and Materials Engineering, Vilnius Gediminas Technical University, 10105 Vilnius, Lithuania; 2 Department of Mechatronics, Robotics and Digital Manufacturing, Vilnius Gediminas Technical University, 10105 Vilnius, Lithuania; 3 Department of Nanotechnology, State Research Institute Centre for Physical Sciences and Technology, 02300 Vilnius, Lithuania; domas.pirstelis@ftmc.lt; 4 Department of Electrical Engineering, Vilnius Gediminas Technical University, 10105 Vilnius, Lithuania

**Keywords:** fuel cells–biofuel cells, electrocatalysis, yeast

## Abstract

This study investigates microbial fuel cell (MFC) performance through the modification of *Saccharomyces cerevisiae* with gold nanoparticles (AuNPs) and polypyrrole (PPy). The yeast/AuNP-modified electrodes generated the highest median current of 2.57 nA, significantly outperforming the yeast/PPy-modified (0.82 nA) electrodes. Power density measurements further confirmed the superior performance of the yeast/AuNP-modified electrodes, showcasing a notable improvement in current densities and power outputs. Yeast/AuNP-modified graphite electrodes produced a higher power density of 22.8 mW/m^2^, while exhibiting a lower current density compared to electrodes modified solely with yeast, which achieved a power density of 5.7 mW/m^2^. These findings highlight the potential of AuNPs in significantly enhancing the electrochemical performance of yeast-based MFCs, providing a promising approach for the development of more efficient bioelectrochemical systems.

## 1. Introduction

The rapidly growing demand for energy resources and the need to monitor environmental pollution have led to the shift to alternative energy sources [1]. Microbial fuel cells (MFCs) can also be considered as an alternative energy source, especially for solving the problem of energy consumption in wastewater treatment plants [2]. They harness the catabolic activity of microorganisms to convert chemical energy into electrical energy, offering a sustainable alternative to conventional fuel cells [3]. Despite having numerous advantages, the primary disadvantage of MFCs is their limited ability to transfer charge through cell walls and membranes, thereby restricting their widespread adoption [4]. Various solutions are used for this purpose. One of them is the use of carbon and metal nanoparticles and electrically conductive polymers in biolayers on the anode and cathode. In this way, an increase in the surface area and conductivity of the active electrode and biocompatibility is achieved [5]. Metal nanocatalysts, such as metal oxides, are considered low-cost alternatives to the commonly used expensive platinum [6,7,8]. In addition, polymeric membranes modified with hydrophilic and antibacterial nanoparticles can increase proton conductivity and reduce biofouling [9]. These improvements may lead to broader applications of microbial fuel cells in energy generation, wastewater treatment, and biosensors.

Similarly to conventional fuel cells, MFCs are composed of an anode and a cathode, typically separated by a selectively permeable membrane [10]. A biofilm forms as a result of microbes attaching to the anode, serving as a catalyst that promotes electricity production [11]. Essential characteristics of anode material are high conductivity, non-corrosiveness, a high surface-to-volume ratio, lack of deposition, high porosity, ease of manufacturing, cost-effectiveness, and scalability [12]. Of these factors, electrical conductivity is most critical, which sets a material apart from other biofilm reactants. Additionally, for effective electrical connections, bacteria need to adhere to the materials. Introducing coatings to these materials also impacts the bacteria’s ability to transfer electrons to the surface through nanowires, mediators, or direct contact [13]. Carbon-based compounds have become highly favored among these materials due to their exceptional efficiency [12,14]. Several studies have investigated the effectiveness of various structures of these graphite-based materials in their interaction with microorganisms during electron transfer processes [15,16].

A key objective of utilizing nanomaterials in anode production is to enhance electron transfer mechanisms between microorganisms acting as biocatalysts in the anode chamber and the material forming the anode, thus improving power output [17]. Nanostructured materials can be applied to alter the surface of an anode electrode made of various materials or used as the primary anode material [18]. Platinum has long been the leading material for oxygen reduction reaction catalysts in microbial fuel cells because of its exceptional catalytic properties. However, its application in large-scale wastewater treatment is largely hindered by its high cost [19]. Their instability as electrocatalysts for the oxygen reduction reaction, coupled with sluggish reaction dynamics and the high cost of platinum, has significantly restricted the broad commercialization of fuel cells utilizing these materials [20,21]. Studies indicate that Pt-based cathodes may represent over 50% of the total capital costs associated with MFC setups, rendering them economically impractical for broad adoption [22]. Gold nanoparticles (AuNPs) are considered suitable for anode modification in MFCs because of their wide range of advantageous qualities, including good biocompatibility, a high surface-to-volume ratio, and enhanced conductivity [23,24,25]. This stands in contrast to other low-cost metal nanoparticles, such as Ag and Cu, which have been shown to exhibit bacterial inactivation and toxicity under certain conditions [26,27]. Additionally, the remarkable conductivity of AuNPs allows them to act as electron receptors [28]. Extensive research has utilized AuNPs to immobilize various redox proteins and enzymes, such as hemoglobin, myoglobin, and horseradish peroxidase, on the electrode surface, facilitating direct electron transfer between proteins [29,30]. Gold nanoparticles have demonstrated their efficacy as electrocatalysts for the oxygen reduction reaction, a process that often serves as the rate-limiting step in microbial fuel cells. Citrate-stabilized AuNPs, as utilized in this study, have demonstrated significant electrocatalytic activity for the oxygen reduction reaction, indicating their potential to improve current generation within MFC architecture [31]. From an economic viewpoint, the expense associated with AuNP synthesis and processing is typically regarded as less burdensome compared to that of Pt. The economic feasibility of utilizing AuNPs is influenced by their sustainable production methods, particularly biological synthesis techniques that limit the use of harmful chemicals and lower production expenses [32,33,34].

Because of its widespread availability, low cost, and ease of harvesting, baker’s yeast (*Saccharomyces cerevisiae* (Sc)) can be advantageous in designing cost-effective biofuel cells. The utilization of *Saccharomyces cerevisiae* in bioelectronics is widespread due to their easy accessibility, their cost-effectiveness, and them having been thoroughly studied as unicellular eukaryotic cells [35]. However, the efficiency of yeast-based biofuel cells is constrained by the insulating properties of the yeast cell wall [36]. To enhance the conductivity of the baker’s yeast cell wall, modifications with carbon-based nanostructured materials [37] and polymers [38,39] have already been conducted. For example, it was proposed that yeast modification with carbon nanotubes can increase the voltage and power generated by the biofuel cell, although in larger quantities, it may harm yeast viability [37]. Another method is the modification of yeast with polypyrrole (PPy), which improves charge transfer efficiency between the yeast and the electrode, although it affects the size of yeast cells by complicating cell proliferation [38]. It was determined that among polymers, polypyrrole exhibits superior biocompatibility with living cells and microorganisms, offering a significant advantage over high-cost carbon nanotubes and modified organic matrices, which possess lower capacitance and redox activity [40,41].

The aim of this study is to investigate the use of gold nanoparticles (AuNPs) to enhance the conductivity of the baker’s yeast (*Saccharomyces cerevisiae*) cell wall.

## 2. Materials and Methods

### 2.1. Materials

Whatman^®^ Nuclepore track-etched membrane (Sigma-Aldrich, Steinheim, Germany) and poly-L-lysine, 9,10-phenantrenequinone, YPD broth, pyrrole (98%), and D-(+)-glucose (99%) were purchased from Merck (Carrigtohill, Ireland), and graphite electrodes and chemicals were purchased from Sigma-Aldrich (Steinheim, Germany). Very popular commercially available bakers’ yeast was purchased from a food supplier, “Dr. Oetker Lietuva” (Vilnius, Lithuania).

Phosphate-buffered solution (PBS, pH 6.8) was prepared by dissolving 0.05 M sodium acetate (CH_3_COONa), 0.05 M disodium phosphate (Na_2_HPO_4_), and 0.1 M potassium chloride (KCl) in distilled water. 9,10-phenanthrenequinone (PQ) was dissolved in 97% ethanol, to a final concentration of 4 mM. Ethanol was purchased from “UAB Vilniaus Degtine” (Vilnius, Lithuania). Additionally, 1 M glucose was prepared by dissolving glucose (≥98%) in PBS. Before investigations, glucose solutions were allowed to mutarotate overnight. (K_3_ [Fe (CN)_6_]) potassium ferricyanide was dissolved in distilled water, at a concentration of 500mM.

Gold nanoparticles, 10 nm diameter, OD 1, with stabilized suspension in citrate buffer (Sigma-Aldrich, Steinheim, Germany), were purchased from Biotecha (Vilnius, Lithuania).

### 2.2. Yeast Preparation

First, 1 g of YPD broth was mixed with 20 mL distilled water to obtain medium with a YPD broth concentration of 50 g/L. Then, 500 mg of dried yeast was introduced to the prepared suspension. The culture was further grown in a shaking incubator at 200 rpm till yeast reached Logarithmic Phase (20–24 h.) Yeast was harvested by centrifugation at 3000× *g* for 5 min and washed with PBS 3 times. The wet mass was weighed and suspended in PBS to a concentration of 1 g/mL.

**Figure 1:**
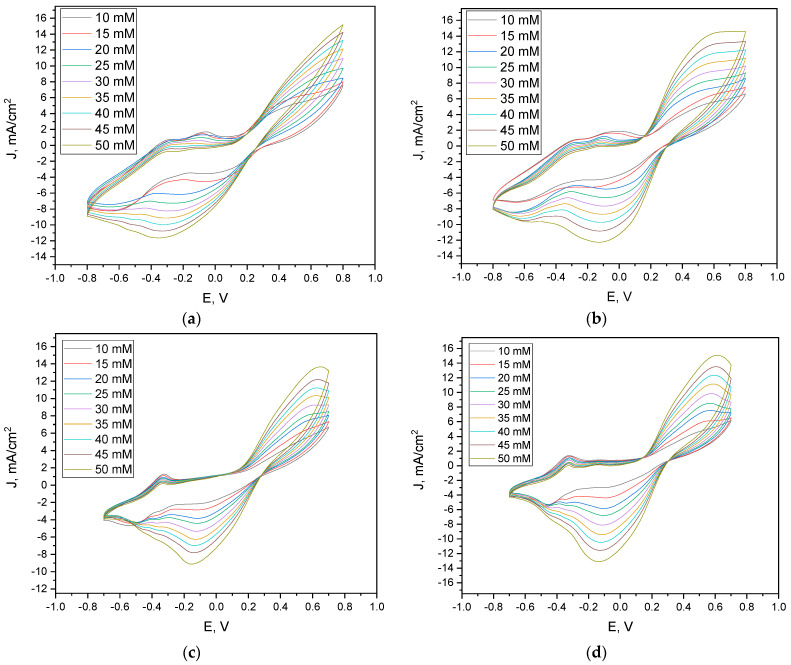
Cyclic voltammograms registered using a (**a**) Sc: Au graphite electrode, (**b**) graphite electrode modified only with yeast, (**c**) Sc: PPy: Au mixture-modified graphite electrode, and (**d**) graphite electrode modified only with PPy-modified yeasts at different potassium ferricyanide (K_3_ [Fe (CN)_6_]) concentrations in the buffer solution. Scan rate of 0.1 V/s and step size of 2 mV were applied.

**Figure 2:**
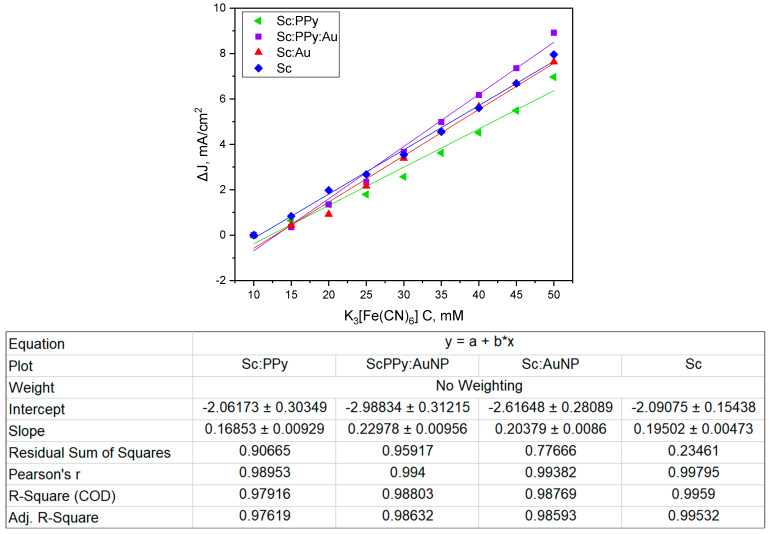
Oxidation peaks of current from the voltammogram as a dependency on potassium ferricyanide (K_3_ [Fe (CN)_6_]) concentration on a yeast/AuNP-modified graphite electrode (Sc: Au), graphite electrode modified only with yeast (Sc), graphite electrode modified only with PPy-modified yeasts (Sc: PPy), and PPy-modified yeast/AuNP mixture-modified graphite electrode (Sc: PPy: Au).

Modification of Saccharomyces cerevisiae with PPy was carried out following a patent recipe [42] with certain modifications implemented. The solution containing 0.3 M pyrrole, 0.4 M potassium ferrocyanide, 1 M glucose, and PBS was used and incubated at 30 °C on an orbital shaker at 250 rpm for 2 h. The cell harvesting and suspension preparation parameters and conditions were the same as those applied for yeast preparation. The effective deposition of PPy was confirmed using electrochemical characterization (Figure 1 and Figure 2), revealing that PPy-modified yeast displayed unique redox activity in contrast to unmodified yeast. Supplementary evidence was presented by SECM analysis, which demonstrated diminished local current aligned with PPy-induced modifications in surface electron transfer characteristics.

### 2.3. Graphite Electrode Preparation

The graphite electrode (rod with 25 mm length, 3 mm diameter, low density, 99.995% trace metal basis) was cut and sanded with sandpaper with five different grinding bead sizes and polished with paper. The prepared electrode was washed with distilled water and 97% ethanol. PQ was immobilized onto the prepared graphite electrode before yeast immobilization with concentration 4 mM, with a 3 µL aliquot. The yeast suspension (1 g/mL) was mixed with the AuNP solution (OD 1) in a 1: 1 volume ratio, and a 4 µL aliquot of this mixture was immobilized on the electrode modified by bakers’ yeast.

### 2.4. Calculations

Electrochemical measurements were evaluated by using Hill’s function:

(1) $$J=\frac{C^{n}}{k^{n}+C^{n}},$$

where *J* is current density, *C* is the concentration of the selected substrate (glucose, PQ, or potassium ferricyanide), *k* is a constant, which is equal to the substrate concentration at which half of the maximal current is observed, and *n* is a Hill coefficient.

### 2.5. Electrochemical Measurements

Electrochemical measurements were performed using a Metrohm µStat-i 400 Potentiostat/Galvanostat (Utrecht, The Netherlands) and DropView 8400M software. All experiments were carried out at ambient temperature (20 °C). The electrochemical cell consisted of three electrodes: a graphite electrode was connected as the working electrode, a platinum electrode as the counter electrode, and a Ag/AgCl (KCl, 3 M) electrode as the reference electrode. All of the components (borosilicate glass titration vessel with a plastic mounting ring and lid, platinum, and 12.5 cm long Ag/AgCl (KCl, 3 M) electrodes) were purchased from Metrohm AG (Herisau, Switzerland). If the concentration of the glucose or other materials was changed during the measurements, this was achieved sequentially by adding said material to the same electrochemical cell.

Ten cyclic voltammetry cycles were carried out for each experiment with a rate of potential from −0.7 V to 0.7 V, a scan rate of 0.1 V/s, and a step size of 2 mV. Only the final cyclic voltammetry cycles were plotted.

Localized electrochemical measurements were performed using Sensolytics Scanning Electrochemical Microscopy (Bochum, Germany) with the Autolab 128 N BiPotentiostat/Galvanostat (Utrecht, The Netherlands). A platinum ultramicroelectrode (UME) with a 10 µm radius (Bochum, Germany) and Rg of 10 was used as the working electrode, a high-surface-area platinum electrode as the counter electrode, and a micro Ag/AgCl (KCl, 3 M) electrode as the reference electrode, purchased from Metrohm AG (Herisau, Switzerland). Before experiments, the UME was cleaned by performing cyclic voltammetry at manufacturer-provided settings in 0.5 M H_2_SO_4_ until no additional peaks were observed. Each horizontal scan was carried out at a 50 µm distance from the surface, with a step size of 20 µm, speed of 20 µm and waiting time of 10 ms, and potential bias at +400 mV.

Power measurements were performed using the Fluke 289 True-RMS Data Logging Multimeter (Fluke, Everett, WA, USA). All experiments were carried out at ambient temperature. The graphite electrode (both modified and unmodified in two different systems) was connected as the anode, and a graphite rod was connected as the cathode.

## 3. Results

Electrochemical behavior of the modified electrodes was evaluated through cyclic voltammetry in the presence of 20 mM glucose and increasing concentrations of potassium ferricyanide (K_3_ [Fe (CN)_6_]), ranging from 10 to 50 mM. All electrode variants showed a concentration-dependent increase in current density, confirming the mediator’s role in facilitating electron transfer.

The yeast/AuNP-modified electrode (Sc: Au, Figure 1a) exhibited cathodic peak potentials shifting from −0.38 V to −0.09 V, while the yeast-only (Sc, Figure 1b) electrode showed narrower peak shifts between −0.13 V and −0.09 V. Electrodes incorporating PPy-modified yeast (Sc: PPy, Figure 1d) and their AuNP composites (Sc: PPy: Au, Figure 1c) demonstrated cathodic peak intervals between −0.16 V and −0.05 V. Anodic peak potentials ranged from 0.38 to 0.44 V (Sc: Au), 0.42 to 0.54 V (Sc), 0.57 to 0.66 V (Sc: PPy: Au), and 0.52 to 0.61 V (Sc: PPy).

Quantitatively, the Sc: Au electrode’s current density increased from 4.5 to 7.0 mA/cm^2^ (at 0.4 V) and −4.2 to −11.5 mA/cm^2^ (at −0.3 V) across the tested mediator range (Figure 1a). In contrast, the Sc electrode exhibited a broader increase—from 5.0 to 13.1 mA/cm^2^ (at 0.5 V) and −4.3 to −12.2 mA/cm^2^ (at −0.15 V) (Figure 1b). The Sc: PPy: Au electrode increased from 5.8 to 13.4 mA/cm^2^ (at 0.6 V) (Figure 1c), while Sc: PPy electrodes reached the highest current values: 14.5 mA/cm^2^ (at 0.55 V) and −12.0 mA/cm^2^ (at −0.1 V) (Figure 1d). These results indicate that PPy-based modifications yielded the highest absolute currents, although not necessarily the most favorable power output, as discussed further below.

Several factors could result in lower current density in a biofuel cell modified by AuNPs. This includes the agglomeration of AuNPs onto cells, which could decrease the available surface area for electron transfer, thereby reducing current density. Additionally, inadequate attachment of AuNPs to the yeast cell wall might lead to fewer available sites for electron transfer, thereby lowering current density. Furthermore, AuNP modification may cause electrode fouling, hindering electron transfer and reducing current density. Moreover, if AuNPs do not enhance the permeability or even compromise the integrity of the yeast cell wall, it may limit electron transfer, resulting in lower current density.

To quantify the influence of potassium ferricyanide concentration on electron transfer efficiency, anodic peak currents obtained from cyclic voltammetry were plotted against mediator concentration (Figure 2). All electrode configurations demonstrated a linear increase in current density with rising concentrations of K_3_ [Fe (CN)_6_] (10–50 mM), consistent with a direct electron transfer mechanism mediated by the redox shuttle. The highest rate of increase was observed for the PPy-modified systems, while the Sc: Au electrode maintained stable and proportional current responses, confirming efficient mediator utilization across all modifications.

To evaluate the bioelectrocatalytic response to substrate availability, cyclic voltammetry was performed using glucose concentrations ranging from 0 to 60 mM in the presence of 30 mM potassium ferricyanide (K_3_ [Fe (CN)_6_]). The selection of 30 mM potassium ferricyanide was based on our experimental findings, which demonstrated a distinct linear correlation between mediator concentration and current density in the 10–50 mM range. The use of 30 mM guaranteed that the mediator concentration was adequate to facilitate efficient electron transfer, all while staying within the linear response range. This enabled us to establish uniform conditions for assessing the impact of glucose concentration while avoiding mediator saturation and excessive reagent consumption. All electrode configurations exhibited a progressive increase in both anodic and cathodic current densities with increasing glucose concentration, indicating enhanced microbial metabolic activity and corresponding electron transfer.

**Figure 3:**
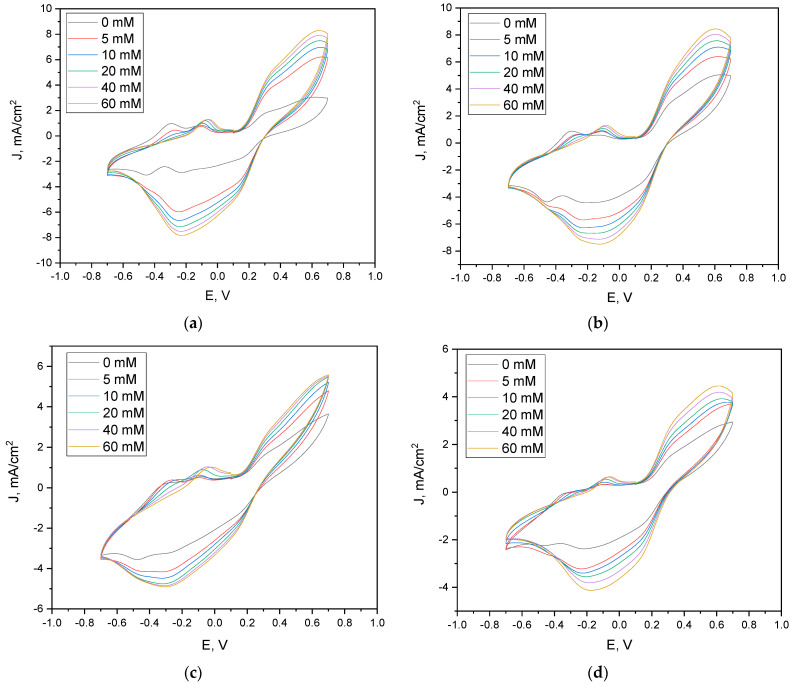
Cyclic voltammograms registered using a (**a**) Sc: Au-modified graphite electrode, (**b**) graphite electrode modified only with yeast, (**c**) Sc: PPy: Au mixture-modified graphite electrode, and (**d**) graphite electrode modified only with PPy-modified yeasts at different glucose concentrations in the buffer solution. Scan rate of 0.1 V/s and step size of 2 mV were applied.

The yeast/AuNP-modified electrode (Sc: Au, Figure 3a) displayed well-defined redox peaks, with cathodic potentials consistently observed at −0.25 V and anodic peaks centered at 0.65 V across all glucose levels. The yeast-only electrode (Sc, Figure 3b) showed similar redox behavior, with slight cathodic shifts from −0.25 V to −0.13 V and anodic shifts from 0.65 V to 0.60 V. Electrodes containing polypyrrole exhibited broader and less defined peaks: the Sc: PPy: Au configuration showed cathodic peaks between −0.23 V and −0.18 V and anodic peaks ranging from 0.57 V to 0.66 V; the Sc: PPy-only electrodes displayed the most negative cathodic shifts (−0.30 V to −0.27 V), with anodic responses consistently at 0.65 V (Figure 3c,d).

Quantitatively, the Sc: Au electrode achieved a current density increase from 3.0 to 8.3 mA/cm^2^ at 0.65 V, while cathodic current increased from −2.9 to −7.8 mA/cm^2^ at −0.25 V (Figure 3a). The yeast-modified electrode exhibited a smaller anodic increase (5.0 to 8.4 mA/cm^2^) and cathodic rise (−4.4 to −7.3 mA/cm^2^) (Figure 3b). Both PPy-containing systems demonstrated reduced electrochemical performance. The Sc: PPy: Au electrode increased from 3.5 to 5.4 mA/cm^2^ (anodic) and from −3.0 to −4.6 mA/cm^2^ (cathodic) (Figure 3c), while the Sc: PPy-only system increased from 2.7 to 4.4 mA/cm^2^ and −2.3 to −3.6 mA/cm^2^ (Figure 3d), respectively.

These results indicate that the Sc: Au electrode not only exhibits the highest electrochemical response but also maintains redox peak stability across the tested glucose concentrations. The enhanced responsiveness and signal clarity suggest that the AuNP modification effectively promotes electron transfer efficiency in yeast-based bioanodes under varying substrate conditions.

**Figure 4:**
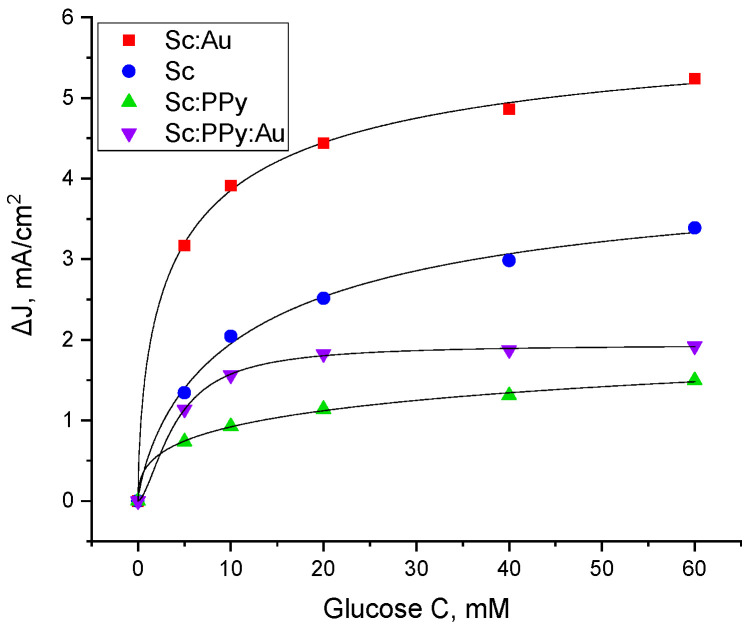
Oxidation peaks of current from the voltammogram as a dependency on potassium ferricyanide (K_3_ [Fe (CN)_6_]) concentration on a Sc: Au-modified graphite electrode, graphite electrode modified only with yeast (Sc), Sc: PPy: Au mixture-modified graphite electrode, and electrode modified with only PPy-modified yeast (Sc: PPy).

To further interpret the electrochemical behavior in response to glucose concentration, the current density at the oxidation peak was modeled using Hill’s equation (Equation (1)). This analysis enabled the evaluation of kinetic parameters and cooperative behavior between the electrode surface and the bioelectrocatalyst (Figure 4).

The Hill coefficient (n) for the Sc: Au electrode was determined to be 0.62, indicating negative cooperativity—wherein the efficiency of charge transfer decreases as substrate concentration increases. A similar trend was observed for the yeast-only electrode (n = 0.78) and the Sc: PPy-modified electrode (n = 0.40), both suggesting a limited synergistic interaction among redox-active sites. In contrast, the Sc: PPy: Au-modified electrode exhibited a Hill coefficient of 1.6, consistent with positive cooperativity, suggesting that electron transfer is facilitated as substrate levels increase. Despite this favorable kinetic profile, the Sc: PPy: Au system did not yield superior current densities, indicating that other factors such as film conductivity or microbial interface quality may restrict overall performance.

The half-saturation constant (k), representing the substrate concentration at which the current reaches half its maximum value, provided insight into reaction kinetics. The Sc: Au electrode demonstrated a k value of 4.61 mM, reflecting rapid redox activity and efficient mediator turnover. The Sc: PPy: Au electrode exhibited a slightly faster response (k = 4.04 mM), whereas the yeast-only electrode responded more slowly (k = 12.8 mM). The slowest kinetics were observed for the Sc: PPy-only electrode (k = 103.2 mM), indicating diminished bioelectrocatalytic efficiency.

These kinetic parameters confirm that the Sc: Au-modified electrode exhibits not only strong electrochemical signals but also favorable reaction kinetics, making it a promising candidate for glucose-driven microbial fuel cell systems. While the Sc: PPy: Au electrode demonstrates fast kinetics and cooperative behavior, its limited current output underscores the importance of optimizing both chemical modification and biological compatibility for improved MFC performance.

The observed discrepancies in ΔJ values between Figure 2 and Figure 4, despite nominally identical experimental conditions (30 mM potassium ferricyanide and 20 mM glucose), can be attributed to the intrinsic variability of biological–electrochemical systems. Each dataset was produced using separately prepared electrodes and yeast samples, which may exhibit modest variations in characteristics such as modification efficiency, biofilm uniformity, or surface coverage. These nuanced changes can affect electron transfer rates and lead to discrepancies in current density, even in otherwise uniform conditions. We acknowledge that this may result in perceived discrepancies between numbers; nonetheless, such volatility is anticipated and does not undermine the credibility of the observed trends. This explanation is provided to emphasize that the data fall within the standard range of experimental variability for biological systems.

**Figure 5:**
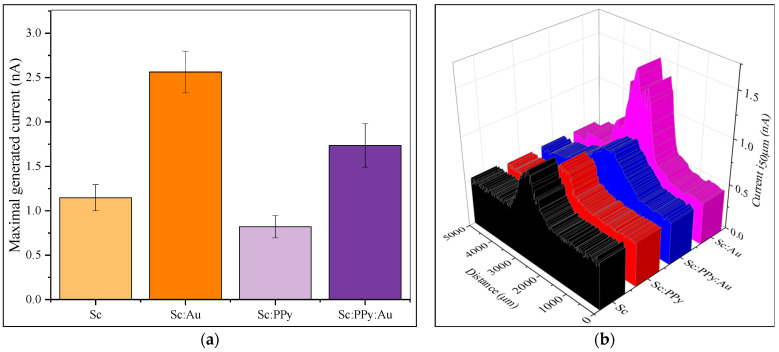
Results from scanning electrochemical microscopy. (**a**) Statistical analysis of gathered data from horizontal scans. (**b**) Horizontal scans of 50 µm above the immobilized yeast sample. Solution contained 30 mM K_3_ [Fe (CN)_6_], 7.5 µM PQ, and 60 mM glucose. Experiments were performed with step 20 µm, speed 20 µm/s, waiting time 10 ms, and potential of +400 mV. Each experiment was repeated five times.

Scanning electrochemical microscopy (SECM) was performed to assess the performance of modified electrodes (Figure 5). Measurement data was recorded in constant-height mode at a 50 µm distance from the surface. To avoid any interferences from the electrode material itself, a non-conductive surface was modified using varying mixtures of baker yeast, and the experimental solution contained 30 mM potassium ferricyanide (K_3_ [Fe (CN)_6_]) as a hydrophilic mediator, 7.5 µM 9,10-phenanthrenequinone as a lipophilic mediator, and 60 mM glucose. Among the tested modifications, the Sc: Au surface generated the highest median current of 2.57 nA, whereas the Sc: PPy-modified surface produced the lowest median current of 0.82 nA. This indicates that AuNPs enhance charge transfer between yeast and the electrode, while PPy impairs this process, as evident by a current reduction of over 1.86 nA. Surfaces modified with Sc: PPy: Au exhibited a median current of 1.74 nA, higher than the Sc: PPy surface but lower than the Sc: Au-modified surface, with a reduction of 0.83 nA on average. These findings suggest a synergistic but incomplete enhancement when combining PPy and AuNP with yeast.

The statistical analysis of the SECM measurements, employing one-way ANOVA and, subsequently, Tukey’s post hoc test, demonstrated that all electrode modifications showed statistically significant differences in local current responses. The Sc: Au-modified surfaces exhibited the highest median currents, greatly exceeding the responses observed in Sc-only, Sc: PPy, and Sc: PPy: Au modifications. Each electrode variant exhibited notable differences from the others, suggesting that both the nature of the modification and the incorporation of AuNPs or PPy have a clear impact on electron transfer efficiency at the microscale. The results indicate that the modification with AuNP significantly improves electron transfer from *S. cerevisiae*, whereas the presence of PPy alone obstructs this process. The findings from SECM enhance the bulk electrochemical data and validate that surface modifications have a direct and measurable impact on localized electron transfer dynamics, which is essential for optimizing MFC electrode performance.

**Figure 6:**
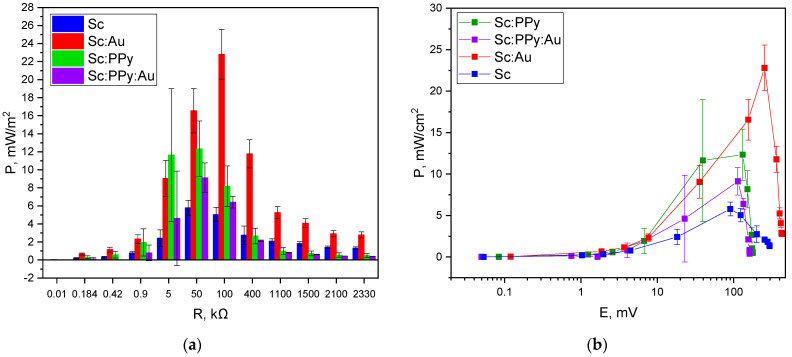
Power density dependence, registered using (**a**) a Sc: Au-modified graphite electrode, graphite electrode modified only with yeast (Sc), graphite electrode modified only with Sc: PPy, and Sc: PPy: Au mixture-modified graphite electrode at 30 mM potassium ferricyanide (K_3_ [Fe (CN)_6_]) and 20 mM glucose concentration in the buffer solution, on applied external load and (**b**) potential. All measurements were performed in two-electrode electrochemical cell.

**Figure 7:**
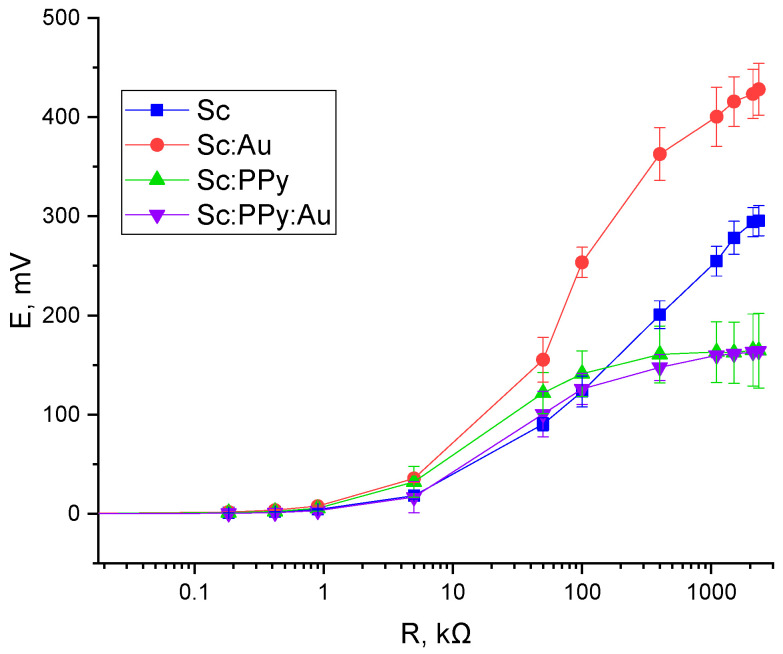
Potential dependence, registered using a Sc: Au-modified graphite electrode, graphite electrode modified only with yeast (Sc), graphite electrode modified only with Sc: PPy, and Sc: PPy: Au mixture-modified graphite electrode at 30 mM potassium ferricyanide (K_3_ [Fe (CN)_6_]) and 20 mM glucose concentration in the buffer solution, on applied external load. All measurements were performed in two-electrode electrochemical cell.

In order to assess MFC performance, a two-electrode-based electrochemical cell was applied, with a (i) Sc: Au-modified graphite electrode, graphite electrode modified only with yeast (Sc), graphite electrode modified only with PPy-modified yeasts (Sc: PPy), or Sc: PPy: Au mixture-modified graphite electrode (ii) and the cathode based on a bare graphite electrode (Figure 6 and Figure 7).

The open circuit potential (OCP) varied significantly depending on the electrode modification. The Sc: Au-modified system exhibited the highest OCP at 455 mV, followed by the yeast-only (Sc) electrode at 312 mV. Electrodes modified with Sc: PPy alone and Sc: PPy: Au composites generated substantially lower potentials of 178 mV and 160 mV, respectively. These results reflect the superior ability of AuNPs to maintain potential stability and promote effective charge separation at the electrode–electrolyte interface (Figure 7).

Maximum power output was determined by measuring current and potential across a range of external loads. The Sc: Au-modified electrode achieved a peak power density of 22.8 mW m^2^ at an external resistance of 100 kΩ. The Sc electrode reached a lower maximum of 5.7 mW m^2^ at 50 kΩ, while the Sc: PPy-modified electrode attained 12.4 mW m^2^ at 50 kΩ. The Sc: PPy: Au composite-modified electrode displayed a peak output of 7.4 mW m^2^, occurring at a much lower resistance of 5 kΩ (Figure 6).

The variation in optimal load and corresponding power density highlights the differences in internal resistance and charge transfer properties among the tested systems. While PPy-modified electrodes exhibited higher current densities during voltammetry, their power output under load was diminished, likely due to increased internal resistance or reduced biocompatibility of the conducting polymer layer. In contrast, the Sc: Au configuration combined high OCP, moderate current response, and optimal resistance matching, leading to the most favorable power performance among all configurations tested.

Statistical analysis of the power measurements using one-way ANOVA with Tukey’s post hoc test revealed that significant differences between electrode modifications emerged predominantly at higher external loads. At 100 kΩ, the Sc: Au-modified electrodes demonstrated significantly higher power output compared to Sc, Sc: PPy, and Sc: PPy: Au electrodes. At 50 kΩ, Sc: Au also showed significantly better performance than Sc and Sc: PPy: Au. In contrast, at lower loads, no statistically significant differences were detected between the different modifications. These results indicate that the enhancement provided by AuNPs is particularly effective under higher-resistance conditions, which are commonly encountered in practical MFC applications. This supports the suitability of AuNP-modified yeast electrodes for systems optimized for high-resistance operation, where maximizing power density and efficiency is critical.

These measurements confirm that AuNPs integrated with *S. cerevisiae* enhance overall electrochemical performance and power output in MFC systems, even when applied to simple graphite electrodes under ambient conditions.

## 4. Discussion

This study demonstrates the enhancement of microbial fuel cell performance via electrode surface modification using *Saccharomyces cerevisiae* and gold nanoparticles. Among the investigated configurations, the Sc: Au-modified graphite electrodes exhibited superior electrochemical behavior, achieving a maximum power density of 22.8 mW/m^2^. This represents a fourfold improvement over unmodified yeast electrodes (5.7 mW/m^2^) and a marked increase compared to electrodes modified with PPy-modified yeast (12.4 mW/m^2^) and Sc: PPy: Au composites (7.4 mW/m^2^). These findings are consistent with previous reports demonstrating the positive role of noble metal nanoparticles in enhancing extracellular electron transfer in MFCs [23,24,25,28].

Electrochemical analysis using cyclic voltammetry revealed that the Sc: Au-modified electrodes achieved current densities up to 8.3 mA/cm^2^ at 0.65 V, with cathodic currents reaching −7.8 mA/cm^2^ at −0.25 V under increasing glucose concentrations. While electrodes modified with PPy-based yeast exhibited slightly higher peak currents, this did not translate into improved power output. This observation aligns with earlier findings that PPy, despite its high conductivity, can interfere with cell proliferation or membrane functionality in microbial systems [38,39]. The diminished performance of Sc: PPy and Sc: PPy: Au electrodes suggests that biocompatibility and biofilm integrity may outweigh raw conductivity in determining net power output.

Scanning electrochemical microscopy (SECM) measurements confirmed the superior electron transfer behavior of Sc: Au-modified biofilms. These surfaces produced the highest median current (2.57 nA), surpassing yeast-only (~1.85 nA), Sc: PPy-modified (0.82 nA), and Sc: PPy: Au (1.74 nA) configurations. These results further support the hypothesis that AuNPs provide a conductive bridge between microbial redox centers and the electrode surface, as previously demonstrated in studies immobilizing redox proteins or enzymes onto AuNP-functionalized substrates [29,30].

Kinetic analysis using Hill’s model yielded a coefficient of n = 0.62 for Sc: Au electrodes, indicating negative cooperativity under increased substrate conditions. In contrast, Sc: PPy: Au electrodes exhibited n = 1.6, indicating positive cooperativity; however, the enhanced transfer kinetics did not result in improved overall performance. These results highlight the complexity of bio-nano interfaces, where faster redox behavior may not always lead to greater energy conversion if other parameters (e.g., microbial activity, interface resistance) are suboptimal.

It is crucial to highlight that although AuNPs greatly improve electron transfer, the selection of *Saccharomyces cerevisiae* is also essential. The resilience, metabolic adaptability, and economic viability of yeast position it as a practical biocatalyst for large-scale MFC systems, particularly when paired with conductive nanomaterials to address its natural electron transfer constraints.

Comparison with literature benchmarks (Table 1) highlights the relative position of this system within the broader field. The power density of 22.8 mW m^2^ exceeds that of simpler carbon systems, such as AuNPs on carbon paper (2.0 mW m^2^) [43], or graphene-modified carbon cloth with air cathodes (2.85 mW m^2^) [44]. These systems, although accessible, are often limited by low surface area or insufficient catalytic activity [6,12,13]. In contrast, high-performance MFCs using catalytically active or 3D substrates have achieved outputs of 264–2771 mW m^2^, such as in systems based on hydrogel–graphene oxide composites or Au-MBA-functionalized carbon felt [45,46,47,48,49]. However, these approaches often involve high-cost materials or complex fabrication protocols.

Notably, the present results are comparable to systems using graphite rod electrodes modified with AuNPs and PPy, which have achieved up to 61.1 mW m^2^ [51]. It is important to note that the power density of 61.1 mW/m^2^ reported by Kižys et al. [51] was obtained using real wastewater as the substrate, whereas our measurements were conducted in a defined buffer solution containing glucose and potassium ferricyanide. The difference in substrate complexity and microbial diversity likely contributes to the higher power output reported by Kižys et al., and therefore, direct comparison with our system should be made with this context in mind. Interestingly, a nearly identical configuration tested in this study (Sc: Au on graphite rod) produced 22.8 mW^2^, while the addition of PPy led to a reduction in performance to 7.4 mW m^2^. These results reinforce prior conclusions that AuNPs are more compatible with yeast-based systems than conductive polymers, especially under mild, biologically relevant conditions [35,41].

Upon examining the microorganisms utilized in the studies outlined in Table 1, it is evident that systems leveraging naturally electrogenic bacteria like *Shewanella* or mixed anaerobic consortia generally attain superior power densities, attributed to their inherent extracellular electron transfer capabilities. For instance, systems that employ diverse microbial consortia have achieved power densities as high as 920 mW/m^2^ [42]. Conversely, *S. cerevisiae*, although not inherently electroactive, offers high accessibility, cost-effectiveness, and ease of handling without the need for strict anaerobic conditions, rendering it appealing for scalable and economical applications. The reduced power output noted in yeast-based systems, exemplified by the 22.8 mW/m^2^ attained in this study, highlights these biological constraints while also emphasizing the promise of surface modifications—such as AuNP integration—to help close the performance disparity between eukaryotic and prokaryotic systems.

This study contributes to the advancement of accessible and efficient microbial fuel cell technologies by demonstrating that electrode modification with gold nanoparticles can significantly enhance bioelectrochemical performance while maintaining system simplicity. The Sc: Au-modified electrodes yielded increased power density without reliance on expensive catalysts or complex fabrication processes. The results underscore that power output is governed not solely by maximum current density, but by the overall efficiency of charge transfer across the bioelectrode interface, including factors such as biocompatibility, electron mediation, and surface interaction dynamics.

This study concentrates on *S. cerevisiae*; however, subsequent investigations should consider analogous nanomaterial modifications for naturally electrogenic bacteria like *Shewanella oneidensis*, *Geobacter sulfurreducens*, or economically viable mixed microbial consortia, which could demonstrate inherently enhanced extracellular electron transfer abilities.

It is important to recognize that although potassium ferricyanide functioned well as a redox mediator in this investigation, its use in large-scale or continuous MFC systems is constrained by environmental and safety issues. In practical scenarios like wastewater treatment, it is crucial to utilize sustainable and non-toxic alternatives. Possible alternatives consist of naturally occurring redox mediators such as flavins and quinones generated by specific electrogenic bacteria, or humic substances sourced from organic matter, all of which have demonstrated potential in improving electron transfer while ensuring environmental compatibility [52,53].

To further improve system performance, future work should investigate the influence of electrode surface area, nanomaterial morphology, and composite architecture on microbial adhesion and electron transfer efficiency. The incorporation of hierarchical or conductive carbon nanostructures in combination with AuNPs may offer synergistic benefits. Additionally, comprehensive evaluation of operational stability, electrode fouling, and nanoparticle retention under continuous working conditions will be essential for assessing the long-term feasibility of these systems in practical applications, including wastewater treatment and biosensing. Future studies ought to concentrate on (i) the integration of electrogenic bacteria or mixed microbial consortia with nanoparticle-modified electrodes, (ii) the development and testing of environmentally benign redox mediators or mediator-free configurations, and (iii) the optimization of MFC architecture, which includes electrode material design, surface area maximization, and flow system engineering, to improve scalability and efficiency.

## Figures and Tables

**Table 1:** Comparison of anode, anode modification, cathode, and power density for several MFCs.

Anode Material	Nanoparticles	Cathode Material	Microorganism Used	Power Density, mW/m^2^	Ref.
Carbon cloth	Graphene	Carbon cloth	*Pseudomonas aeruginosa*	52.5	[50]
Carbon cloth	Graphene microsheet	Air cathode	*Escherichia coli* B	2.85	[44]
PANIPAM hydrogel	Graphene oxide	Carbon cloth	*Escherichia coli* ATCC	264	[45]
Carbon paper	TiO_2_-Au	Carbon paper	*Shewanella loihica* PV-4	497	[46]
Carbon felt	Au-MBA	Carbon felt	*Saccharomyces cerevisiae*	2771	[47]
Carbon paper	AuNPs	Carbon paper/Pt	*Saccharomyces cerevisiae*	2	[43]
Carbon felt	HNO3	Carbon paper	*Saccharomyces cerevisiae*	450	[48]
Carbon felt	PDA/PPY	Graphite rod	Mixed microbial consortium derived from secondary anaerobic digester sludge	920	[49]
Graphite rod	AuNPs/PPy	Graphite rod	*Saccharomyces cerevisiae*	61.1	[51]
Graphite rod	AuNPs	Graphite rod	*Saccharomyces cerevisiae*	22.8	This work

## Data Availability

The original contributions presented in this study are included in the article. Further inquiries can be directed to the corresponding author.

## Abbreviations

The following abbreviations are used in this manuscript:

MFC	Microbial fuel cell
AuNPs	Gold nanoparticles
PPy	Polypyrrole
PQ	9,10-phenanthrenequinone
SECM	Scanning electrochemical microscopy
Sc	*Saccharomyces cerevisiae*
